# Managing resources in NHS dentistry: using health economics to inform commissioning decisions

**DOI:** 10.1186/1472-6963-11-138

**Published:** 2011-05-31

**Authors:** Richard D Holmes, Jimmy Steele, Catherine E Exley, Cam Donaldson

**Affiliations:** 1 Centre for Oral Health Research, School of Dental Sciences, Newcastle University, Framlington Place, Newcastle upon Tyne, NE2 4BW, UK; 2 Institute of Health and Society, Newcastle University, Baddiley-Clark Building, Richardson Road Newcastle upon Tyne NE2 4AX UK; 3 Yunus Centre for Social Business and Health, Glasgow Caledonian University, 3rd Floor, Buchanan House, Cowcaddens Road, Glasgow, G4 0BA, UK

## Abstract

**Background:**

The aim of this study is to develop, apply and evaluate an economics-based framework to assist commissioners in their management of finite resources for local dental services. In April 2006, Primary Care Trusts in England were charged with managing finite dental budgets for the first time, yet several independent reports have since criticised the variability in commissioning skills within these organisations. The study will explore the views of stakeholders (dentists, patients and commissioners) regarding priority setting and the criteria used for decision-making and resource allocation. Two inter-related case studies will explore the dental commissioning and resource allocation processes through the application of a pragmatic economics-based framework known as Programme Budgeting and Marginal Analysis.

**Methods/Design:**

The study will adopt an action research approach. Qualitative methods including semi-structured interviews, focus groups, field notes and document analysis will record the views of participants and their involvement in the research process. The first case study will be based within a Primary Care Trust where mixed methods will record the views of dentists, patients and dental commissioners on issues, priorities and processes associated with managing local dental services. A Programme Budgeting and Marginal Analysis framework will be applied to determine the potential value of economic principles to the decision-making process. A further case study will be conducted in a secondary care dental teaching hospital using the same approach. Qualitative data will be analysed using thematic analysis and managed using a framework approach.

**Discussion:**

The recent announcement by government regarding the proposed abolition of Primary Care Trusts may pose challenges for the research team regarding their engagement with the research study. However, whichever commissioning organisations are responsible for resource allocation for dental services in the future; resource scarcity is highly likely to remain an issue. Wider understanding of the complexities of priority setting and resource allocation at local levels are important considerations in the development of dental commissioning processes, national oral health policy and the future new dental contract which is expected to be implemented in April 2014.

## Background

The resources associated with providing NHS dental services are sizable. In December 2010, a Department of Health publication outlining proposals for piloting a future new dental contract in England, stated that NHS dentistry accounted for almost £3 billion of public expenditure (including patient charges) [1]. Primary Care Trusts (PCTs) in England currently manage devolved finite resources as a result of the new General Dental Services (nGDS) contract introduced in April 2006. Until that time, PCTs had little control over the national NHS dental budget as resources were held centrally and administered largely through dentists submitting their requests for payment on a fee-per-item and/or capitation basis. Many stakeholders incorrectly viewed the centrally held NHS dental budget as 'non-cash limited' and arguably, few would have referred to NHS dental services as particularly 'resource constrained'.

The nGDS contract in England and Wales charged PCTs (and Local Health Boards in Wales) with the responsibility for ensuring that appropriate dental services were developed which were tailored to the needs of local populations. Until 2006, local health organisations had never before been placed in such a prime position through which to shape local NHS dental services using local commissioning. Despite this, significant concerns have been expressed regarding the great variation in the commissioning skills between PCTs. The Health Select Committee Report on Dental Services published in July 2008 criticised the commissioning arrangements for NHS dentistry by highlighting:

*'In-house commissioning skills vary greatly between PCTs. As the Minister acknowledges, too many PCTs are not doing a good job...' *[2].

The Government response to the Health Committee Report acknowledged that work needed to involve addressing the continued variability in the quality of dental commissioning [3]. At the time of hearing the evidence, the Health Select Committee was informed that the role of the PCT was 'currently very weak' [4], and a national survey by the Patients Association published in March 2008 similarly criticised PCTs for a lack of creativity in their dental commissioning arrangements [5].

The 2007 Annual Health Check undertaken by the Healthcare Commission in England, revealed that almost forty percent of PCTs scored 'fair' or 'weak' in their use of resources [6] and in relation to dental services the need for improved guidance on best practice has been highlighted [5]. The introduction of a vision for World Class Commissioning [7] together with the reforms led by Lord Darzi [8], firmly placed PCTs at the time, as the 'leaders of the local NHS'. As a consequence, this study proposal was designed around two PCTs who effectively manage local NHS dental services as one commissioning organisation, and a large secondary care teaching dental hospital.

Primary dental care appeared as a national priority in the Operating Framework for the NHS in England 2009/10 [9] and the Framework referred to improving NHS dental services in a number of key areas:

*'While progress in some priorities is commendable, a lot more needs to be done to improve access to dentistry, as well as the quality of care and oral health in the community' *[9].

Research conducted by the authors has highlighted a lack of clarity with regard to how some PCTs structure their commissioning processes in order to ensure the efficient use of finite resources for NHS dentistry [10,11]. Indeed, the earlier Operating Framework for the NHS in England 2008/09 similarly made specific reference to the commissioning of dental services:

'*PCTs need to ensure robust commissioning strategies for primary dental services, based on assessments of local needs and with the objective of ensuring year-on-year improvements in the number of patients accessing NHS dental services*'[12].

The recent Government reviews and publications on NHS dental services together with the research team's earlier studies [10,11], collectively suggest that PCTs may benefit from further guidance and support in their dental commissioning responsibilities. As a consequence, these organisations might then be supported to use finite resources more efficiently and equitably. Resources do not simply refer to financial inputs but they include consideration of staffing and workforce requirements. In a recent 2010 'local commissioning survey' conducted by the British Dental Association, it was reported that seventy-four percent of dental commissioning leads in PCTs across England felt they needed additional support in their dental commissioning teams [13]. Eighty-one PCTs participated in the survey which equates to a 53% response rate.

The 2009/10 NHS Operating Framework in England placed emphasis upon a need to review dental commissioning strategies in order that transparent and open procurement processes exist [9] and the current 2011/12 Operating Framework also calls upon PCTs to commission improvements in access to NHS dentistry and improve efficiency through effective contract management [14]. In response to the collective concerns regarding the local commissioning of NHS dental services, the research team propose a pragmatic economics-based approach to structure the dental commissioning process. The rationale behind proposing this approach is to expressly consider the key economic principles 'opportunity cost' and 'the margin' to determine whether resources for dentistry may be re-allocated (within the service) to maximise efficiency.

There are several economic approaches that could be applied in the study (for example cost-benefit analysis CBA, cost-effectiveness analysis CEA, and cost-utility analysis CUA). However, research has highlighted challenges to their application in practice [15-23]. A key issue for healthcare decision-makers is that technically sound health economics methods often cannot reflect the driving complexities of the commissioning process [24]. Programme budgeting and marginal analysis (PBMA) draws upon the same theoretical principles as the economic analyses listed above. However, it is less constrained by having to pre-define the measure of outcome to be used and, indeed, would permit multiple outcomes into the evaluation framework. Thus, it also provides a more flexible framework which can be applied to existing commissioning processes, akin to the use of traditional decision analytic approaches [25]. PBMA has been applied successfully in Australia where the process allowed immediate decisions to be made regarding the local priorities for dentistry [26]. The application of PBMA in other health systems has similarly shown positive impacts to priority setting and in the allocation of scarce health care resources [27].

### Aim of the study

The aim of this study is to develop, apply and evaluate an original economics-based framework built upon a PBMA approach, to assist commissioners in their management of finite resources for dental services. The study will draw upon key economic principles associated with PBMA to inform and guide commissioners in their delivery of appropriate dental services which are tailored to the needs of local populations.

### Action Research Question

How can health economics improve the commissioning of NHS dental services for the benefit of patients and local populations?

## Methods/Design

We propose an action research approach [28] which will apply mixed methods to record the opinions, successes and challenges facing stakeholders involved with decision-making for NHS dental services in both primary and secondary care settings in northern England. The study will also document the stages involved in the application of a pragmatic economics-based framework with which to structure the decision-making process. The rationale for applying action research as a scientific approach is threefold. First, to involve NHS staff and other participants as 'co-researchers' working closely *with *the research study (rather than simply doing research *on *them), second, to identify organisational factors (e.g. management structures and workforce) within the PCTs involved that may impact upon the commissioning of local oral health care services, and third, to determine whether action research can bring about a degree of change within these NHS organisations [29] as a result of using health economics in the commissioning process.

The importance of participant involvement in the research process was identified in a NHS Health Technology Assessment published in 2001 which included a systematic review relating to action research [30]. The subsequent report acknowledged the importance of participant involvement in the research process and recognised how this approach may increase active engagement of users in NHS services. Of direct relevance to our proposed study, the report suggested that action research could be used for the development of knowledge and understanding in relation to informed decision-making [30].

### Data collection

Data generated by this study will be collected in the field by one member of the research team (RH). Initially, the study will explore the views of stakeholders regarding issues facing NHS dental services from the perspectives of each group as an in-depth scoping exercise. A combination of semi-structured interviews with PCT staff and NHS dentists together with focus groups comprising service-users, will document participants' responses according to a pre-piloted topic guide. It is estimated that approximately twenty semi-structured interviews with NHS professionals and up to four focus groups with service-users is likely to achieve data saturation in the settings identified. Each interview and focus group will follow a topic guide which will be modified and updated in response to feedback received from participants throughout the research study. It is anticipated that each interview and focus group will last approximately 60 minutes. Advisory panel meetings are a core component of PBMA approaches and these will comprise up to three representatives from each stakeholder group. A number of these panel meetings will be convened during the study in order to agree local priorities for NHS dental services, to finalise the prioritisation criteria for use in the decision-making process and to consider relevant data sources with which to inform potential resource reallocation within the existing dental budget.

**Figure 1:**
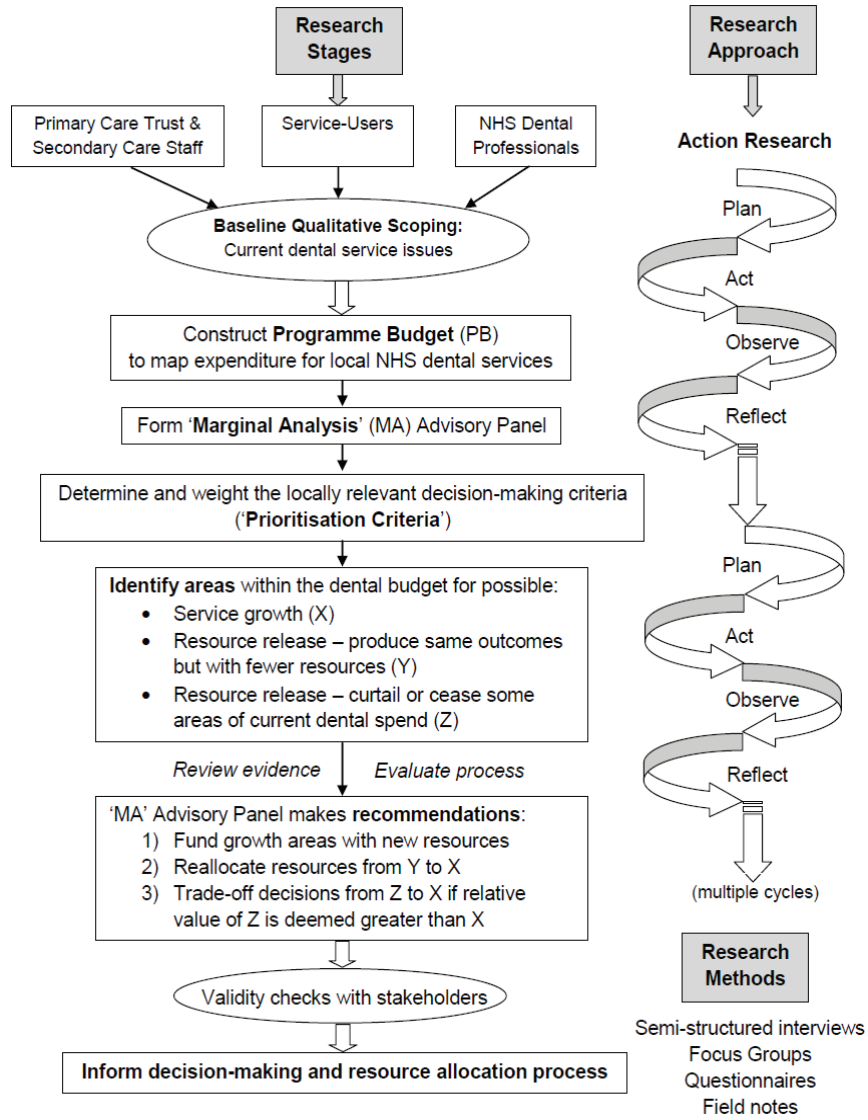
Research stages using action research and mixed methods

Figure 1 outlines an anticipated order of the research methods and stages in the nominated primary and secondary care settings. The diagram also outlines the key stages involved in a generic programme budgeting and marginal analysis exercise and is based upon earlier work published by one of the research team (CD) [31].

Following a baseline qualitative assessment of current dental issues facing stakeholders, two inter-related PBMA case-studies will be conducted - the first will be based in primary dental care and the second in a secondary care (dental teaching hospital) environment. Although the details of each process will be specific to each case-study, the generic process for each would be as follows. A detailed map of current dental expenditure within the NHS organisations will be produced by the Principal Investigator working with the PCT and hospital, and this will be distributed to all participant groups for comment. This will form the Programme Budget (PB) and it will aim to clarify how resources are currently spent on NHS dentistry. The study will them move into the Marginal Analysis (MA) phase whereby resources are considered for reallocation in order to maximise oral health 'per pound spent'. During the initial stages of the research study, all participants will be asked to identify local priorities for investment and disinvestment in NHS dental services before decision-making criteria are agreed and weighted by each group. All NHS professionals will be invited to nominate areas within the local dental budget for investment and disinvestment through the use of an anonymous, customised postal questionnaire. Service-users will undertake the same process within the baseline focus group meetings.

A number of dental business cases (or proposals) will be agreed by the advisory panel which are based upon the emergent priorities for local NHS dental services and a decision-making process using PBMA will be operationalised in order to rank the proposals according to the decision-making (or 'prioritisation') criteria.

Throughout this process, the P.I. will act as a facilitator at advisory panel meetings and will work alongside fellow participants within the multiple action research cycles of 'plan', 'act', observe' and 'reflect'. Additional focus groups and semi-structured interviews will document participants' experiences of their involvement with the dental decision-making process. Focus groups and semi-structured interviews will be digitally audio-recorded and professionally transcribed verbatim for subsequent analysis. Written questionnaires and voting forms will be devised to both weight and rank the proposed dental programs under consideration, alongside the agreed prioritisation criteria. Data analysis will therefore include both qualitative and simple quantitative techniques.

Participants will consider written evidence (both clinical and financial) in support of each proposal. The data and inputs required to inform the decision-making process will be decided by the panel. Examples may include summaries of clinical dental guidelines, research papers detailing the clinical effectiveness of interventions and the associated costs and benefits of the business cases under consideration. For each proposal, the facilitator will prepare a 'panel approved' business pro forma. The pro forma will include sufficient detail to enable all members of the advisory panel to ultimately award a score against the agreed prioritisation criteria. Advisory panel meetings will encourage discussion amongst the group, they will provide an opportunity to consider additional scientific evidence and they will directly involve representatives from each of the three stakeholder groups in a local priority setting and resource allocation exercise.

### Study sample

The study will use a purposive sampling strategy to ensure that the stakeholder groups' views are represented in the data. Service-user representation will be sought by formally approaching the Chair of the PCT's Local Involvement Network (LINk) inviting members to participate in the study. Dental practitioners will first be identified and approached via the PCT's Dental Practice Advisor (DPA) who will also contact the Chair of the Local Dental Committee (LDC). PCT staff will include dental commissioners and public health practitioners who will be contacted via letter directly by the PI inviting them to participate.

Inclusion criteria comprise adults over the age of 18 years who live and/or work within the geographic area of the selected NHS organisations and who would fall into one of the three stakeholder groups (local service-user/NHS dentist/PCT staff). Exclusion criteria include children and teenagers aged 17 years and below, participants unable to consent for themselves and adults who do not speak English.

As is common in qualitative research, the final sample size will be determined by the need to achieve data saturation [32]. However, as a guide in the planning phase, and based upon the research team's earlier work, it is envisaged that the study will recruit approximately fifty participants in total from the three stakeholder groups identified in both the primary and secondary care-based case studies

An overarching 'advisory panel' will be convened at an early stage during the research study. This panel will provide balanced representation from each stakeholder group. Although this is a commonly-recognised step in the PBMA approach, it is common for multi-disciplinary and multi-functional groups to be set up within health organisations to review service in specific areas (in this case, dentistry). In this sense, PBMA merely seeks to build on what already happens in such organisations.

### Data analysis and interpretation

Data collection and analysis will occur concurrently using the constant comparative method [33] in order to incorporate the responses of participants into topic guides. Professionally produced audio transcripts from the recorded semi-structured interviews and focus groups will be returned to the P.I. for qualitative analysis. Each transcript will be labelled with a unique participant identifier to ensure that the identity of participants remains confidential. Thematic analysis will be used throughout and the data will be managed manually using a framework approach [34]. The validity of data interpretation will be strengthened through independent coding and analysis by at least two members of the research team (RH and CE). Regular feedback to participants of the results generated to date, will attempt to ensure that the main themes and findings are interpreted and reported accurately. The facilitator will use the beginning of each focus group and advisory panel meeting to present the emergent themes and outcomes generated by earlier sessions. Participants will then be asked for their views and comments in order to verify that the data and meeting outcomes are a true record. This reciprocity is an inherent component of an ethical action research approach [35].

The research team will adhere to COREQ (Consolidated criteria for reporting qualitative research) criteria [36] for reporting qualitative research in papers which arise from this study. COREQ comprises a 32-item checklist to assist researchers in their reporting of study parameters. The three domains which form the COREQ checklist include: the research team and reflexivity; study design; and analysis and findings [36].

### Ethical approval

The study has approval from County Durham and Tees Valley 2 Research Ethics Committee [Ref: 10/H0908/9] and NHS Research Governance approval from the participating NHS organisations involved in primary care. Further ethical reviews will be submitted as 'substantial amendments' as the study evolves in response to the views of participants. The principal investigator (RH) holds an honorary NHS contract and is the only member of the research team to have direct access to patients and NHS staff in this study.

Participant data will be stored confidentially by the principal investigator in accordance with the Data Protection Act 1998 and local NHS protocols. Written consent will be taken from each participant on enrolment and a unique identifier code will protect each participant's anonymity alongside published verbatim quotes taken from the transcripts.

Management for the recruitment of service-users will be devolved to the Chair or organiser of the local engagement groups (e.g. LINks). This will mean that the research team do not need to contact members of the public directly (thus reducing any potential for inadvertent coercion), nor will the research team need to store personal data such as the home addresses of members of the public.

### Study limitations

The research is based upon a series of in-depth qualitative case studies in primary and secondary care NHS organisations in northern England. The organisational structure within each setting may contain unique aspects from which it may not be possible to generalise to other NHS settings in England. However, in defence of the action research and qualitative approaches selected, it was considered appropriate to ground the study firmly within existing NHS organisations in order to explore and document the complexities surrounding dental priority setting and decision-making. The recent government Spending Review and the White Paper 'Equity and Excellence: Liberating the NHS' has announced the proposal to abolish PCTs from April 2013 [37]. This has led to demonstrable flux and the initiation of transitional arrangements within the PCTs identified. As a consequence, the level of engagement with the study by time-constrained PCT staff is likely to be a real challenge for the research team. In light of workforce cuts already evident within these PCTs, the researchers will endeavour to fit the study around existing dental business to reduce any additional burden upon participants. Similarly, the research team will try to ensure that panel meetings do not always occur during normal business hours so that NHS dentists are not prevented from conducting their normal clinical duties. Despite government plans to abolish PCTs, the commissioning of NHS dental services at local or regional levels is almost certain to continue within the context of resource scarcity. This study will first explore the current status of dental commissioning within the NHS organisations involved and it will investigate how PBMA may act as a framework to structure decision-making processes.

NHS dental services are arguably in need of a range of new clinical outcome measures with which to measure oral health improvement across local populations. Within a resource scarce environment decisions still need to be made. New dental business cases prioritised for implementation as a result of this study may require several years for their clinical effects to be observed in the local population. This time delay will mean that the impact of service changes or preventive or clinical interventions will require detailed follow up over a number of years after the end of this study.

## Discussion

The protocol outlines a study which is of direct and immediate relevance to patients, the public, health professionals and commissioners of NHS dental services. With almost £3 billion of public expenditure currently spent on NHS dental services in England and widespread criticism regarding the variability of dental commissioning, it is timely for research to investigate how we may improve the process in order to use scarce resources more efficiently. The key issue relates to whether we can further maximise the oral health of populations with the resources currently available to local NHS commissioning organisations. In order to improve oral health (and indirectly the general health) of populations, one research direction may be to propose a move away from historic funding allocations, to an oral health service which is built upon local oral health needs and with the combined views and priorities of stakeholders included in the commissioning process. We propose that at the heart of this process should be the recognition of resource scarcity and that managing health needs requires decisions to be made within existing constraints.

Our earlier research conducted as a precursor to this study suggested that there is a real potential for PCTs to be managing dental resources sub-optimally [11]. For example, in NHS dentistry it is known that historically, money has followed activity (dental treatment), not patients' needs [38]. A pragmatic economics-based approach built upon the principles of PBMA may inform and improve our current commissioning arrangements and assist in the realignment of resources to benefit patients and the public.

This research is timely as it seeks to explore the complexities and processes associated with priority setting and resource allocation in order to maximise oral health 'per pound spent'. The study will provide analysis of the complexities involved with decision-making for NHS dental services and determine the value of an economics-based framework with which to structure the commissioning process.

The study aims to address a fundamental question - how best to allocate finite resources for NHS dentistry at local levels? Through the use of action research as an inclusive research approach, the study will seek to involve stakeholders through a series of advisory panel meetings, focus groups and semi-structured interviews in order to weight a number of agreed prioritisation criteria which will be used to score submitted business proposals for developing local NHS dental services. The findings from the study will be important for policy makers to consider when assessing the structure of new commissioning organisations linked to the next NHS dental contract in England and Wales which is anticipated in 2014.

## List of abbreviations

DPA: Dental Practice Advisor; LDC: Local Dental Committee; LINk: Local Involvement Network; LREC: Local Research Ethics Committee; nGDS: New general dental services (introduced in 2006); NHS: National Health Service; NIHR: National Institute for Health Research; PAR: Participatory Action Research; PBMA: Programme Budgeting and Marginal Analysis; PCT: Primary Care Trust; PI: Principal Investigator; SHA: Strategic Health Authority

## Competing interests

The authors declare that they have no competing interests.

## Authors' contributions

RH, CD and JS were involved in the research study design and NIHR funding application. RH drafted this manuscript and is the sole investigator with responsibility for data collection. CE was involved in qualitative data analysis. All authors were equally responsible for revising this manuscript and they approve the content of the final draft.

## Authors' information

RH is a dentist undertaking academic specialist training in dental public health. He holds a Doctoral Research Fellowship from the National Institute for Health Research hosted by Newcastle University (2009-2012). CD holds the Yunus Chair in Social Business and Health at Glasgow Caledonian University and is a National Institute for Health Research Senior Investigator. CE is Senior Lecturer in Medical Sociology, Newcastle University. JS holds the Chair in Oral Health Services Research, Newcastle University and led the 2009 Review of NHS dental services in England.

## Pre-publication history

The pre-publication history for this paper can be accessed here:

http://www.biomedcentral.com/1472-6963/11/138/prepub
